# Ethnopharmacology- and Chemotaxonomy-Guided Evaluation of *Apuleia leiocarpa* Bark and Leaf Extracts against Oropouche, Mayaro, Chikungunya, and Zika Viruses

**DOI:** 10.1021/acsomega.5c10741

**Published:** 2026-03-12

**Authors:** Maria Rosilda Valente de Sarges, Maria Clara Machado Oliveira, Mariana Pereira de Carvalho, Marília Bueno da S Menegatto, Ariane Coelho Ferraz, Sônia das Graças Santa Rosa Pamplona, Wandson Braamcamp Pinheiro, Cintia Lopes B. Magalhães, Milton Nascimento da Silva, Paulo Wender P. Gomes, Consuelo Yumiko Yoshioka e Silva

**Affiliations:** † Amazon Integrated Metabolomics Center, Institute of Exact and Natural Sciences, 37871 Federal University of Pará, Belém 66075-110, Brazil; ‡ Institute of Health Sciences, Pharmaceutical Sciences Post-Graduation Programme, Federal University of Pará, Belém 66075-110, Brazil; § Institute of Exact and Natural Sciences, Federal University of Pará, Belém 66075-110, Brazil; ∥ Biology Sciences Post-Graduation Program, 28115 Federal University of Ouro Preto, Ouro Preto 35400-000, Minas Gerais, Brazil; ⊥ School of Pharmacy, Federal University of Ouro Preto, Ouro Preto 35400-000, Minas Gerais, Brazil; # Biotechnology Post-Graduation Program, Federal University of Ouro Preto, Ouro Preto 35400-000, Minas Gerais, Brazil

## Abstract

*Apuleia leiocarpa* (Vogel) J.F. Macbr. (Fabaceae) is traditionally used to treat fever and inflammatory symptoms, supporting an ethnopharmacological rationale for antiviral investigation. In addition, the chemotaxonomic context of the genus *Apuleia*, characterized by the prevalence of flavonoids and triterpenoids typical of Fabaceae, provides a complementary framework for the selection of this species. Guided by ethnopharmacological use and chemotaxonomic evidence, this study evaluated the *in vitro* antiviral activity of ethanolic and aqueous extracts obtained from the leaves and stem bark of *A. leiocarpa* against Mayaro, Chikungunya, Zika, and Oropouche viruses. Chemical characterization of the extracts was performed using liquid chromatography–tandem mass spectrometry combined with computational metabolomics tools, including molecular networking and mass spectral database searches. Flavonoids, triterpenoids, and phenolic acids were putatively annotated in the extracts, and their presence was interpreted in the context of antiviral activities previously reported for these chemical classes, providing a qualitative framework for the observed antiviral responses. All extracts exhibited antiviral activity against at least one of the viruses tested. Notably, the ethanolic stem bark extract showed potent activity against Oropouche virus, with an EC_50_ value of 14.85 μg/mL and a selectivity index of 51.50. These results indicate that the ethanolic bark extract of *A. leiocarpa* represents a promising and renewable source of natural antiviral leads.

## Graphical abstract

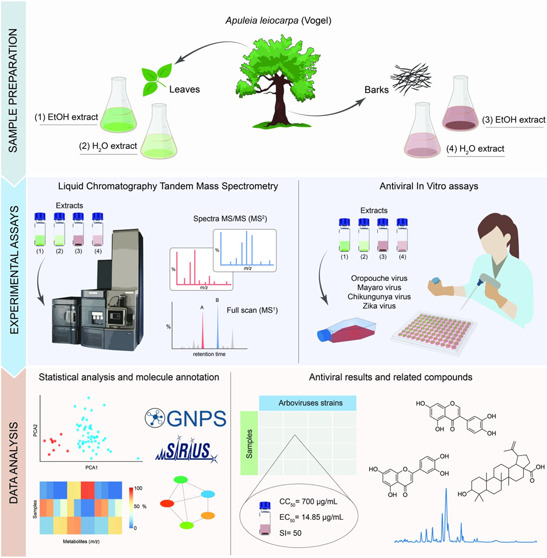

## 1 Introduction

The lack of clinically approved antiviral therapies for emerging arboviruses limits therapeutic options. Despite recurrent outbreaks and sustained transmission in tropical and subtropical regions, clinical management remains largely supportive. Currently, there are no licensed antiviral agents available for most clinically relevant arboviral infections.^1–3^

Ecological conditions in the Amazon basin support the persistence of vectors and the sustained transmission of Oropouche virus (OROV), Mayaro virus (MAYV), Chikungunya virus (CHIKV), and Zika virus (ZIKV).^2,4,5^ Infection by these arboviruses presents as an acute febrile illness with inflammatory manifestations and, in some cases, neurological complications,^1,2^ which reinforces their public health significance in tropical regions.

The remarkable chemical diversity of plant species provides a wide range of secondary metabolites that can be evaluated for biological properties. Historically, this diversity has positioned natural products as one of the most productive sources of lead compounds in drug discovery, including antiviral agents.^6–10^ Using structured criteria to select species increases the probability of identifying antiviral activity in extract-based screening, especially when integrating chemical diversity with ethnopharmacological and taxonomic knowledge.^11^

*Apuleia leiocarpa* (Vogel) J. F. Macbr. (Fabaceae), has been traditionally used by indigenous communities, including the Chácobo people, to treat fevers and inflammation. Since the symptoms of these conditions overlap with those of arboviral infections, it is worthwhile to evaluate the antiviral properties of its extracts. Furthermore, ethnopharmacological knowledge can inform species selection by linking empirical therapeutic practices to biological effects that can be tested in experiments. This strengthens hypothesis-driven natural product research.^11,12^

The Dialioideae subfamily of the Fabaceae family comprises taxa known for producing flavonoids, phenolic derivatives, and triterpenoids. *Apuleia leiocarpa* belongs to this lineage.^13^ The antiviral and anti-inflammatory properties of compounds within these chemical classes have been demonstrated in experimental settings, including interference with viral entry, replication, and host inflammatory pathways.^14–17^ These results suggest that a chemically informed approach to antiviral research aligns with the taxonomic context of this species.

High-resolution LC–MS/MS provides detailed chemical information about secondary metabolites found in botanical extracts. It supports identifying the main compound classes, even when prior isolation is not possible,^18,19^ enabling comprehensive metabolomic profiling of complex mixtures. When used alongside in vitro antiviral assays, this analytical approach correlates chemical composition with observed biological responses at the extract level.^20,21^ This facilitates interpretation of bioactivity within a systems-level framework rather than attributing it to isolated compounds.

We evaluated the antiviral activity of ethanolic and aqueous extracts from the bark and leaves of *A. leiocarpa* in vitro against OROV, MAYV, CHIKV, and ZIKV. Of these viruses, Oropouche virus has recently gained particular epidemiological relevance in the Americas, underscoring the importance of identifying novel antiviral candidates.^2,4,5^ We characterized their chemical composition using metabolomic analysis based on LC–MS/MS. Considering the ethnopharmacological context and chemotaxonomic positioning, this study explores the antiviral potential of plant-derived extracts within a framework of rational selection. This addresses the urgent need for novel therapeutic leads against emerging arboviruses.^2,5^

Within this framework, the present study aimed to perform an ethnopharmacology- and chemotaxonomy-guided evaluation of ethanolic and aqueous extracts obtained from the bark and leaves of *A. leiocarpa*. The antiviral activity of these extracts was assessed in vitro against four clinically relevant arboviruses—OROV, MAYV, CHIKV, and ZIKV—and their chemical composition was characterized using LC–MS/MS–based metabolomic profiling. By integrating traditional knowledge, taxonomically informed chemistry, and antiviral screening, this work seeks to contribute to the identification of plant-derived extracts with potential relevance for the development of novel antiviral agents.

## 2 Methods

### 2.1 Botanical Material and Extraction

Leaf and stem bark samples of *Apuleia leiocarpa* were collected in Belém, Pará, Brazil (1°27′01″ S; 48°25′10″ W). Botanical authentication was performed prior to analysis, and a voucher specimen (IAN 202191) was deposited at the EMBRAPA–Eastern Amazon herbarium. Authorization for access to Brazilian genetic heritage was obtained through SISGEN (A57C662).

The plant material was dried at 45 °C until constant weight and milled using a Pulverisette 14 ball mill (Fritsch, Idar-Oberstein, Germany) to obtain powders with particle sizes between 60 and 100 μm.

Ethanolic extracts were prepared by maceration at room temperature. For each plant part, 100 g of powdered material was extracted with 1 L of ethanol (1:10, w/v) for two consecutive 24 h cycles, followed by vacuum filtration.

Aqueous extracts were prepared by decoction (bark; 100 g in 1 L of ultrapure water, boiled for 10 min) or infusion (leaves; 100 g in 1 L of hot ultrapure water for 20 min), followed by filtration.

### 2.2 Liquid Chromatography-Tandem Mass Spectrometry Analysis

Metabolic profiling was performed by LC–HRMS using a Xevo G2-S QTof mass spectrometer (Waters Corp., USA) equipped with an electrospray ionization (ESI) source operating in negative ion mode (*m*/*z* 50–2000). Leucine–enkephalin was continuously infused as a lock-mass reference to ensure mass accuracy during acquisition. Instrument control and data acquisition were performed using MassLynx 4.1 software.

Chromatographic separation was achieved on a BEH C18 column (50 mm × 2.1 mm, 1.7 μm) maintained at 40 °C. The mobile phase consisted of water (A) and acetonitrile (B), delivered at a flow rate of 300 μL min^–1^ using a linear gradient from 10% to 100% B over 18 min, followed by column washing and re-equilibration.

Sample solutions (2 mg mL^–1^) were filtered through 0.22 μm membranes prior to injection (2 μL). Instrumental conditions were selected based on previously optimized workflows for plant metabolomic profiling.

### 2.3 Data Processing and Feature Extraction

Raw LC–MS/MS data were processed using MZmine 4.0 for feature detection,^22^ chromatographic deconvolution, isotopic grouping, alignment, and export of spectral information. Feature detection parameters were adapted from previously validated metabolomic workflows.^23,24^

Detailed processing parameters are provided in the Supporting Information (Table S1) to ensure methodological reproducibility while maintaining conciseness in the main text. Only features associated with MS/MS spectra were retained for subsequent analysis.

The curated feature table and corresponding MS/MS spectra were exported in .mgf and .csv formats for molecular networking analysis.

### 2.4 Feature-Based Molecular Networking

Feature-based molecular networking (FBMN) was performed using the Global Natural Products Social Molecular Networking (GNPS) platform.^25^ The processed feature table and MS/MS spectral files generated in MZmine were submitted for spectral similarity analysis and network construction following established GNPS workflows.^26^

In the resulting networks, nodes represent precursor ions and edges correspond to cosine similarity relationships between MS/MS fragmentation spectra. Spectral connections were established using a minimum cosine score of 0.7 with at least six matched fragment ions.

Metadata describing the relative ion abundance across extracts were incorporated to facilitate comparative visualization of chemical features. Molecular networks were visualized and explored using Cytoscape 3.10.2.^27^

The GNPS job is publicly accessible at: https://gnps.ucsd.edu/ProteoSAFe/status.jsp?task=b1d9e21d146b44b89267dd3d72b7f9cf

This approach enables the clustering of structurally related metabolites and supports annotation of natural product families based on shared fragmentation patterns.

### 2.5 Antiviral Analysis

#### 2.5.1 Cell Lines and Viruses

Vero cells (ATCC CCL-81) were maintained in Dulbecco’s Modified Eagle Medium (DMEM) supplemented with 5% fetal bovine serum and standard antimicrobial agents at 37 °C in a humidified 5% CO_2_ atmosphere.

The arboviruses evaluated in this study were OROV, MAYV, CHIKV, and ZIKV. Viral strains and propagation procedures followed previously described laboratory protocols and strain characterizations for these arboviruses.^28–31^ Viral stocks were titrated prior to experimental use.

#### 2.5.2 Cytotoxicity Assays

Cytotoxicity was evaluated using an MTT-based cell viability assay with minor modifications from previously described protocols.^32,33^ Vero cells were seeded at a density of 5 × 10^4^ cells per well in 96-well plates and incubated overnight.

Cells were subsequently exposed to serial dilutions of the extracts for 48 h. Cell viability was quantified by measuring absorbance at 490 nm.

The concentration required to reduce cell viability by 50% (CC_50_) was determined by nonlinear regression analysis using GraphPad Prism software.

#### 2.5.3 Antiviral Activity Assays

Antiviral activity was evaluated using cell-based infection models adapted from previously validated experimental systems for arboviruses.^34–37^ Vero cells were infected at a multiplicity of infection (MOI) of 1, and extract concentrations below the CC_50_ were added simultaneously with viral inoculation.

Following incubation periods specific for each virus (48–72 h), cell viability was determined using the MTT assay. The effective concentration required to maintain 50% viability of infected cells (EC_50_) was calculated by nonlinear regression.

Antiviral selectivity was expressed as the selectivity index (SI), calculated as CC_50_/EC_50_.

## 3 Results

### 3.1 Metabolite Annotation

Mass spectrometry data were used to characterize metabolites by employing molecular networking and GNPS library searching, which resulted on annotation of four proanthocyanidins, one flavan-3-ol, eight flavones, three flavanones, ten methoxylated flavonols, and four triterpenes (Table 1). All annotated compounds were carefully assessed to have acceptable mirror plots between the experimental and reference spectra. The metabolite assignment was based on two factors: (1) Cosine score between experimental and reference MS/MS spectra retrieved from GNPS libraries; (2) in absence of reference library matches, literature MS/MS spectra were used to support metabolite annotation. A detailed metabolite characterization including fragments (MS/MS) explanation and mechanisms can be found in the Supporting Information.^38–43^

**Table 1:** Annotated Compounds in Extracts of Leaves and Stem Barks from *A. leiocarpa* ^a^

peak	RT (min)	molecular formula	[M – H]^−^ (*m*/*z*)	experimental	theoretical	error (ppm)	product ions MS/MS	putative compound	reference spectrum ID	extract
*Proanthocyanidins*										
1	0.57	C_30_H_26_O_12_	[M – H]^**–**^	577.1353	577.1346	1.21	125, 161, 205, 245, 289, 339, 407	procyanidin B1	CCMSLIB00012176075	EEBAl
										EELAl
										AEBAl
										AELAl
2	1.16	C_30_H_26_O_12_	[M – H]^**–**^	577.1349	577.1346	0.51	125, 161, 203, 245, 289, 407, 425, 451	procyanidin B2	CCMSLIB00000222141	EELAl
										AEBAl
										AELAl
3	1.78	C_45_H_38_O_18_	[M – H]^**–**^	865.1993	865.1980	1.50	125, 243, 287, 289, 407, 425, 575, 695	procyanidin C	CCMSLIB00010128636	EELAl
										AEBAl
										AELAl
5	2.18	C_45_H_38_O_18_	[M – H]^**–**^	865.1995	865.1980	1.73	125, 243, 287, 289, 407, 425, 575, 695, 713	procyanidin C1	CCMSLIB00012079549	EELAl
										AEBAl
										AELAl
*Flavan-3-ol*										
4	2.10	C_15_H_14_O_6_	[2M – H]^**–**^	579.1494	579.1503	1.55	109, 125, 151, 179, 203, 205, 221, 245	epicatechin	CCMSLIB00011430118	EEBAl
										EELAl
										AEBAl
										AELAl
*Flavones*										
6	2.43	C_21_H_20_O_11_	[M – H]^**–**^	447.0930	447.0927	0.67	133, 163, 175, 285, 297, 311, 327, 339, 357, 429	homoorientin	CCMSLIB00004696807	EEBAl
										EELAl
										AELAl
7	2.71	C_26_H_28_O_14_	[M – H]^**–**^	563.1402	563.1401	0.17	293, 311, 323, 413, 431	isovitexin 2-O-arabinoside	CCMSLIB00000846353	EEBAl
										EELAl
										AEBAl
										AELAl
8	2.87	C_21_H_20_O_10_	[M – H]^**–**^	431.0978	431.0978	0	269, 283, 311, 323, 341	Isovitexin	CCMSLIB00004696020	EEBAl
										EELAl
										AEBAl
										AELAl
9	2.99	C_28_H_32_O_14_	[M – H]^**–**^	591.1711	591.1714	0.50	283, 295, 324, 325, 427, 445, 471	2-O-rhamnosyl-swertisin	CCMSLIB00000077222	EEBAl
										EELAl
										AEBAl
										AELAl
13	4.84	C_15_H_10_O_6_	[M – H]^**–**^	285.0393	285.0399	2.10	107, 133, 151, 175, 199, 217, 241, 267	Luteolin	CCMSLIB00004691240	EEBAl
										AEBAl
15	5.83	C_15_H_10_O_6_	[M – H]^**–**^	285.0400	285.0399	0.35	151, 199, 217, 241, 257	Isoluteolin	CCMSLIB00004691240	EEBAl
										AEBAl
21	7.23	C_18_H_16_O_7_	[M – H]^**–**^	343.0815	343.0818	0.87	226, 242, 270, 285, 298, 313, 328	Eupatilin	CCMSLIB00004683815	EEBAl
										EELAl
										AEBAl
										AELAl
24	8.09	C_17_H_14_O_6_	[M – H]^**–**^	313.0710	313.0712	0.63	255, 270, 283, 298	velutin	CCMSLIB00005777937	EEBAl
										EELAl
										AEBAl
										AELAl
*Flavanones*										
10	3.07	C_21_H_22_O_11_	[M – H]^**–**^	449.1098	449.1084	3.11	107, 125, 151, 285, 303	(neo)astilbin	CCMSLIB00004707540	EEBAl
										AEBAl
11	3.17	C_21_H_22_O_11_	[2M **–** H]^**–**^	899.2274	899.2246	3.11	107, 125, 151, 285, 303	astilbin	CCMSLIB00003136267	EEBAl
										AEBAl
12	3.38	C_21_H_22_O_11_	[M – H]^**–**^	449.1090	449.1084	1.33	107, 125, 151, 285, 303	(iso)astilbin	CCMSLIB00004707540	EEBAl
										AEBAl
*Methoxy flavonols*										
14	5.26	C_19_H_18_O_9_	[M – H]^−^	389.0868	389.0873	1.28	153, 177, 183, 213, 241, 248, 285, 287, 315, 327, 341, 357, 359, 373, 375	5-O–Demethylapulein	[^16^]	EELAl
										AELAl
16	6.02	C_17_H_14_O_7_	[M – H]^**–**^	329.0659	329.0661	0.60	199, 243, 271, 299, 314	4′,5,7-trihydroxy-3,6-dimethoxyflavone	CCMSLIB00004718271	EEBAl
										EELAl
										AELAl
17	6.18	C_20_H_20_O_9_	[M – H]^**–**^	403.1024	403.1029	1.24	165, 211, 271, 298, 315, 327, 341, 373	apulein	[^16^]	EELAl
										AELAl
18	6.35	C_19_H_18_O_8_	[M – H]^**–**^	373.0926	373.0923	0.80	165, 207, 287, 297, 315, 326, 343, 358	5-O-methyloxyayanin-A	[^16^]	EEBAl
										EELAl
										AELAl
19	6.38	C_18_H_16_O_8_	[M – H]^**–**^	359.0763	359.0767	1.11	258, 286, 301, 312, 314, 329, 344	jaceidin	CCMSLIB00004718287	EEBAl
										EELAl
										AEBAl
										AELAl
20	6.71	C_18_H_16_O_8_	[M – H]^**–**^	359.0756	359.0767	3.06	299, 312, 314, 327, 344	apuleidin	[^16^]	EEBAl
										EELAl
										AEBAl
										AELAl
22	7.58	C_18_H_16_O_8_	[M – H]^**–**^	359.0760	359.0767	1.94	221, 243, 267, 289, 299, 311, 327, 344	oxyayanin-A	[^16^]	EEBAl
										EELAl
										AEBAl
										AELAl
23	7.69	C_19_H_18_O_8_	[M – H]^**–**^	373.0917	373.0923	1.60	257, 285, 300, 315, 328, 343, 358	chrysosplenetin	CCMSLIB00005724488	EEBAl
										EELAl
										AEBAl
										AELAl
25	8.58	C_20_H_20_O_9_	[M – H]^**–**^	403.1035	403.1029	1.48	216, 231, 255, 271, 287, 298, 315, 327	apuleirin	[^16^]	EELAl
										AELAl
26	8.59	C_18_H_16_O_7_	[M – H]^**–**^	343.0813	343.0818	1.45	186, 198, 214, 226, 242, 254, 270, 298	ayanin	[^16^]	EEBAll
										EELAl
										AELA
*Triterpenes*										
27	13.75	C_39_H_54_O_6_	[M – H]^**–**^	617.3846	617.3842	0.64	117, 133, 145, 161, 423, 439, 455, 573	2-O-*p*-coumaroyl alphitolic acid	CCMSLIB00003740028	EEBAl
										EELAl
										AEBAl
28	13.94	C_30_H_48_O_3_	[M – H]^**–**^	455.3529	455.3525	0.87	288, 321, 360, 413, 437	betulinic acid	[^17^]	EEBAl
										EELAl
										AEBAl
29	15.38	C_39_H_56_O_5_	[M – H]^**–**^	603.4057	603.4049	1.32	133, 135, 161, 179	3-O-Caffeoyl-betulin	CCMSLIB00011906774	EEBAl
30	15.75	C_39_H_56_O_5_	[M – H]^**–**^	603.4048	603.4049	0.16	133, 135, 161, 179	Isomer 3-O-Caffeoyl-betulin	CCMSLIB00011906774	EEBAl

a Abbreviations: RT: retention time, ppm: part per million, ID: identity, EEBAl: ethanolic extract of the bark; EELAI: ethanolic extract of the leaves; AEBAl: aqueous extract of the bark; and AELAI: aqueous extract of the leaves.

Figure 1 provides a comparative analysis of the molecular profiles from different extracts and parts of *A. leiocarpa*, emphasizing their distinct chemical compositions and potential bioactive compounds. The Principal Component Analysis (PCA) plot reveals a clear clustering of leaves and stem barks, underscoring their unique metabolic signatures based on the explained variance of 83.40%. Furthermore, the distinct separation between ethanolic and aqueous extracts highlights the significant impact of solvent selection on the metabolites extracted, showcasing the importance of extraction methods in phytochemical studies.

**Figure 1:**
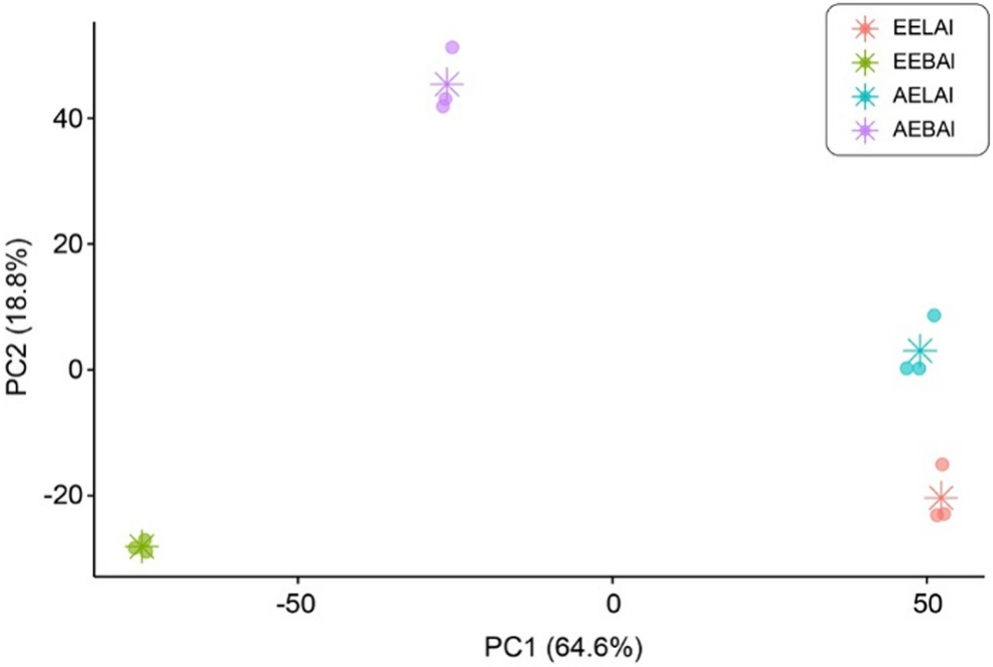
Principal component analysis. PCA shows the spatial distribution between extracts from stem barks and leaves of *A. leiocarpa* (*p* < 0.002). EEBAl: ethanolic extract of the bark; EELAI: ethanolic extract of the leaves; AEBAl: aqueous extract of the bark; and AELAI: aqueous extract of the leaves. Asterisks in the PCA figure indicate group centroids.

Figure 2a presents a heatmap illustrating the normalized peak areas of 150 metabolites with VIP scores >1.0 retrieved from the PLS-DA model across different extract types. Each row represents an individual metabolite, while columns correspond to distinct extracts. The color gradient (blue: lower abundance; red: higher abundance) highlights differences in relative metabolite abundance associated with tissue type and extraction solvent, revealing clear patterns of metabolite distribution among the samples.

**Figure 2:**
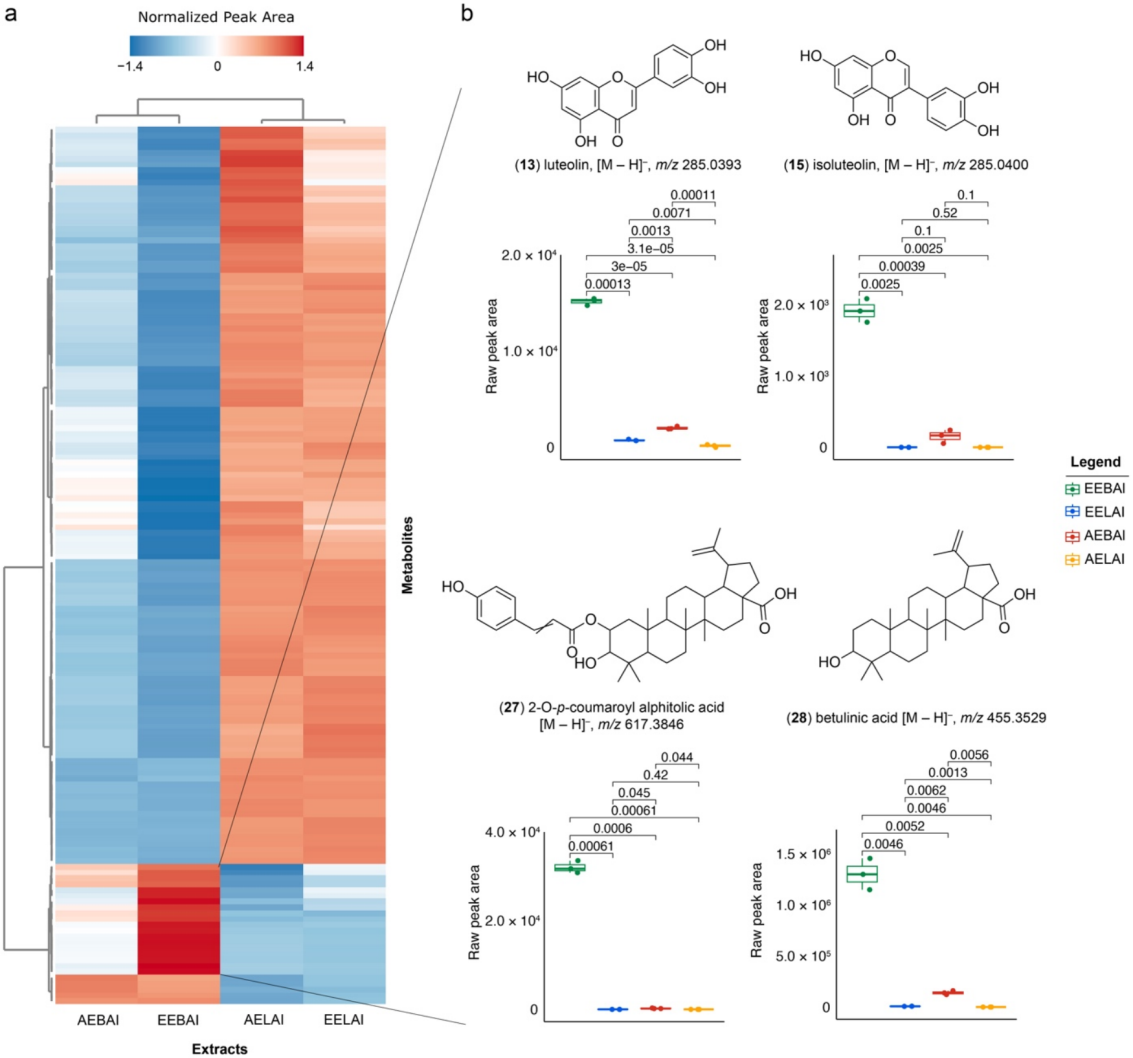
Metabolomics analysis of extracts from *A. leiocarpa*. (a) Heatmap displaying the top 150 features (*m*/*z*) with VIP scores >1.00, derived from paired PLS-DA models. Metabolite annotation was performed using GNPS libraries. (b) Boxplots showing metabolites with high relative abundance in the ethanolic extract of *A. leiocarpa* stem bark, retrieved from the EEBAl: ethanolic extract of the bark; EELAI: ethanolic extract of the leaves; AEBAl: aqueous extract of the bark; and AELAI: aqueous extract of the leaves. A *p*-value <0.05 indicates statistically significant differences across comparisons.

Figure 2b shows boxplots of the relative abundance of four representative metabolites: luteolin ([M – H]^−^, *m*/*z* 285.0393), isoluteolin ([M – H]^−^, *m*/*z* 285.0400), 2-O-*p*-coumaroyl alphitolic acid ([M – H]^−^, *m*/*z* 617.3846), and betulinic acid ([M – H]^−^, *m*/*z* 455.3529). These metabolites were selected based on their high VIP scores, relatively higher abundance trends in the most active extract, and confident or putative annotation supported by MS/MS data and chemotaxonomic relevance. Metabolite identification confidence was assigned in accordance with the Metabolomics Standards Initiative (MSI) guidelines. Betulinic acid (**28**) was confirmed at MSI Level 1 using an authentic standard, whereas the other metabolites were annotated at MSI Level 2 based on accurate mass, MS/MS fragmentation, and chromatographic behavior. Their relative abundance trends in the ethanolic stem bark extract are presented for comparative and exploratory purposes only and should be interpreted as correlation-based, without implying causal relationships or validation as antiviral markers.

### 3.2 Cytotoxicity of the Extracts

The cytotoxicity test allowed the determination of the cytotoxic concentration (CC_50_) of the extracts, defined as the concentration capable of reducing cell viability by 50%. The EEBAl extract had a mean CC_50_ of 764.45 μg/mL with a standard deviation of 16.83, while AEBAl had a CC_50_ of 706.61 μg/mL with a standard deviation of 3.90. On the other hand, EELAl and AELAl had concentrations higher than 500 μg/mL as described in Table 2. Concentration–response curves are presented in Supporting Figure 1.

**Table 2:** Values of the Cytotoxic Concentrations (CC_50_) Presented by the Extracts^a^

cytotoxicity	
extract	CC_50_ (μg/mL)
EEBAl	764.45 ± 16.83
EELAl	>500
AEBAl	706.61 ± 3.90
AELAl	>500

a EEBAl: ethanolic extract of the bark; EELAI: ethanolic extract of the leaves; AEBAl: aqueous extract of the bark; and AELAI: aqueous extract of the leaves.

### 3.3 Antiviral Activity

The effective concentration of each extract for 50% of the cells (EC_50_) and the selectivity index (SI) are shown in Table 3. The EEBAl extract showed the most promising results, as it was able to protect infected cells at low concentrations (Figure 3a), resulting in high selectivity index (SI). For instance, EEBAl against OROV showed an EC_50_ of 14.85 μg/mL (Figure 3b) and an SI of 51.50. The same extract against MAYV showed an EC_50_ of 36.52 μg/mL and an SI of 20.93. Against CHIKV, the EC_50_ was 65.03 μg/mL and the SI was 11.76. And against ZIKV the EC_50_ was 57.70 μg/mL and SI 13.25. On the other hand, the EELAl extract required higher concentrations to achieve cellular protection, as it showed an EC_50_ of 82.20 μg/mL and an SI of 6.08 in cells infected with OROV. Against MAYV it gave an EC_50_ of 77.13 μg/mL and SI 6.48. Also, against CHIKV it showed an EC_50_ of 97.13 μg/mL and SI 5.15. The extract also showed activity against ZIKV with an EC_50_ of 99.82 μg/mL and SI 5.01.

**Figure 3:**
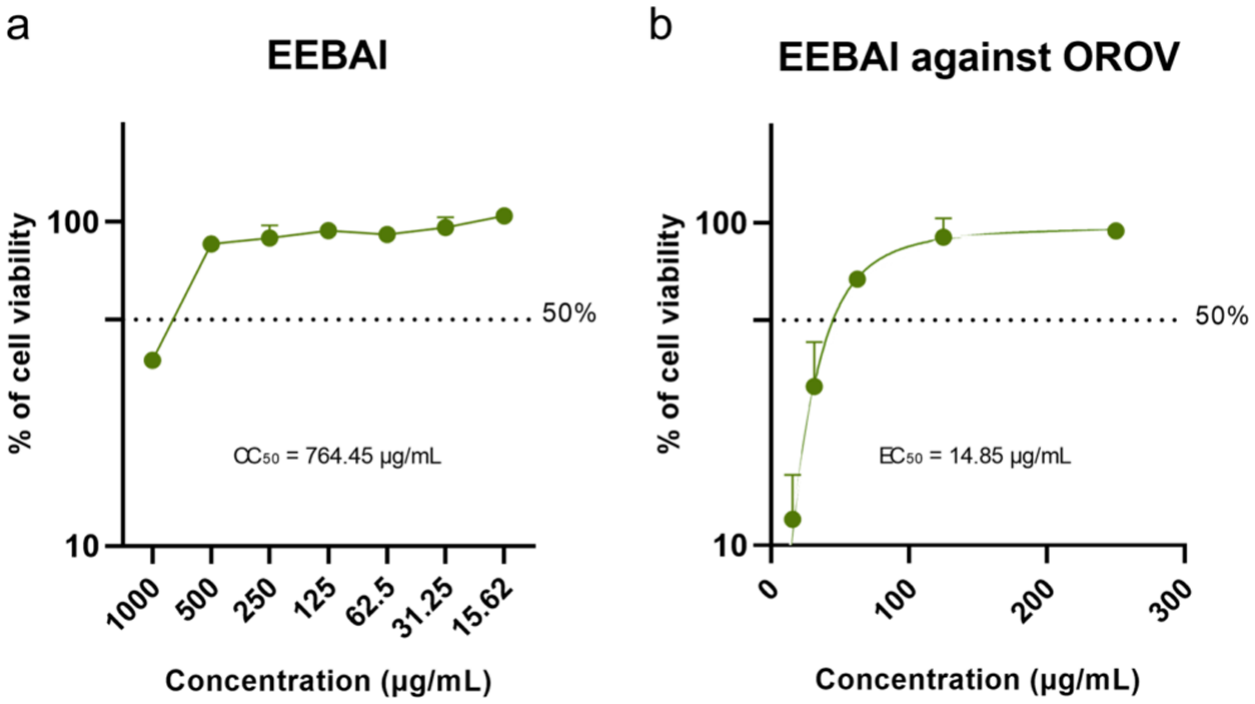
Antiviral activity of the ethanolic extract of the bark from *A. leiocarpa* against Oropouche virus (OROV). (a) Cytotoxicity was assessed after 48 h of treatment with different concentrations of the extract. (b) Antiviral activity was evaluated after 48 h of OROV infection with concomitant treatment with different concentrations of EEBAl. For both assays, cell viability was measured using the MTT (3-(4,5-dimethylthiazol-2-yl)-2,5-diphenyltetrazolium bromide) viability assay.

**Table 3:** Effective Concentrations of the Extracts to 50% Infected Cells and Selectivity Index of the Extracts to the Arboviruses Described in This Study (OROV, MAYV, CHIKV, and ZIKV)^a^

	anti-OROV		anti-MAYV		anti-CHIKV		anti-ZIKV	
extract	EC_50_ (μg/mL)	SI	EC_50_ (μg/mL)	SI	EC_50_ (μg/mL)	SI	EC_50_ (μg/mL)	SI
EEBAl	14.85 ± 6.72	51.50	36.52 ± 2.06	20.93	65.03 ± 7.96	11.76	57.70 ± 13.31	13.25
EELAl	82.20 ± 8.10	6.08	77.13 ± 7.76	6.48	97.13 ± 15.91	5.15	99.82 ± 2.97	5.01
AEBAl	95.04 ± 4.46	7.44	NA	-	151.86 ± 20.60	4.65	174.26 ± 1.13	4.05
AELAl	45.53 ± 1.71	10.98	97.66 ± 20.05	5.12	172.86 ± 26.45	2.89	89.46 ± 9.09	5.59

a NA: the extract did not show activity; EEBAl: ethanolic extract of the bark; EELAI: ethanolic extract of the leaves; AEBAl: aqueous extract of the bark; and AELAI: aqueous extract of the leaves.

Compared to the efficacy of the ethanolic extracts, the aqueous extracts were less promising. The activity of AELAl against OROV was more interesting than the action of this extract with the other viruses, for OROV the EC_50_ was 45.53 μg/mL and SI 10.98, for MAYV the EC_50_ was 97.66 μg/mL and SI 5.12, for CHIKV the EC_50_ was 172.86 μg/mL and SI 2.89 and for ZIKV the EC_50_ was 89.46 μg/mL and SI 5.59. The results of AEBAl were considered less effective compared to the other extracts as it was inactive against MAYV and larger amounts of the extract were required to protect cells against infection by the other arboviruses evaluated, as against OROV it showed an EC_50_ of 95.04 μg/mL and SI 7.44, CHIKV showed an EC_50_ of 151.86 μg/mL and SI 4.65 and ZIKV showed an EC_50_ of 174.26 μg/mL and SI 4.05.

## 4 Discussion

The antiviral activity of the *A. leiocarpa* extracts showed promising results, especially the ethanolic extract of the bark (EEBAl), which showed high antiviral activity (EC_50_) against different strains of infected cells. Therefore, low concentrations of extracts were required to inhibit ZIKV, MAYV, OROV, and CHIKV in infected cells. In addition, the selectivity index (SI) against all arboviruses tested was greater than 10, particularly against OROV, where the SI was as high as 51.50. Literature data emphasize that an SI greater than 10 can be considered safe and with potential for drug development.^10,37^ The SI represents the correlation between the cytotoxic concentration and the effective concentration in 50% of the cells and is therefore important for the evaluation of effective and safe compounds in a given therapeutic window, thus aiding in the selection and development of new drugs.^37,44^

The integration of mass spectrometry data with molecular network analysis and spectral searching using the GNPS platform enabled the annotation of 30 compounds from the ethanolic and aqueous extracts of *A. leiocarpa* stem bark and leaves. Notably, several annotated compounds have previously been associated with antiviral properties, potentially contributing to the antiviral activity observed in this study. As shown in the heatmap of metabolite abundance (Figure 2a), the ethanolic extract of bark (EEBAl) exhibited a significantly higher concentration of key compounds, including luteolin (**13**), isoluteolin (**15**), 2-O-*p*-coumaroyl alphitolic acid (**27**), and betulinic acid (**28**). These metabolites may play a pivotal role in the pronounced antiviral activity identified in the EEBAl extract.

While these metabolites represent highly promising candidates based on their relative abundance and correlation with antiviral activity, it is important to emphasize that the observed effects are attributed to the crude extract. Such activity may arise from synergistic interactions among multiple constituents or from other minor compounds not highlighted here. Therefore, definitive proof of principle will require bioassay-guided fractionation followed by antiviral testing of isolated pure compounds.

Luteolin (**13**) and isoluteolin (**15**) were first reported in *A. leiocarpa*. Luteolin has been reported to have antiviral activity and has shown *in vitro* activity against chikungunya virus (CHIKV) by reducing viral mRNA synthesis in infected cells.^15,45^ These isomers have also been evaluated for antiviral activity against other types of viruses, such as *in vitro* activity against all serotypes of dengue virus (DENV), where inhibition of enzymatic activity occurs in a noncompetitive manner, preventing the formation of infectious mature virions; *in vivo* activity has also been demonstrated in mice infected with DENV.^46^ In addition to these studies, luteolin has also been reported to be active against SARS-CoV-2, influenza virus, enterovirus, rotavirus, herpes virus, and respiratory syncytial virus, among others.^45^ 2-O-*p*-coumaroyl alphitolic acid (**27**), reported for the first time in the species studied, has no studies reporting antiviral activity. This compound has been tested *in vitro* for antiplasmodial activity in *Plasmodium falciparum* and antimycobacterial activity against strains of *M. tuberculosis*.^47^ However, once it has the same aglycone of betulinic acid (a known antiviral compound), we cannot discard this compound as a potential antiviral agent.^48^

Betulinic acid (**28**) has already been reported in *A. leiocarpa* ^8^ and was already reported with *in vitro* activity against DENV, CHIKV, and ZIKV.^48,49^ Specifically in DENV, this compound inhibits a postentry stage of the replication cycle, inhibiting viral RNA synthesis and protein production.^48^ In addition, betulinic acid and its analogs show activity against human immunodeficiency virus (HIV).^50,51^ Also, it has also been shown to inhibit hepatitis B virus (HBV), hepatitis D virus (HDV),^52^ and herpes simplex virus types I and II.^53^ Another study investigates the neuroprotective effect of betulinic acid in ZIKV-infected neural progenitor cells, protecting against virus-induced cell death.^49^ Thus, luteolin (**13**), isoluteolin (**15**), 2-O-*p*-coumaroyl alphitolic acid (**27**), and betulinic acid (**28**) could explain the antiviral activity of EEBAI.

Furthermore, EELAl, AELAI, and AEBAl showed antiviral activity, and other annotated compounds are related. For instance, procyanidin B (**1**, **2**) (in which its isomers are reported for the first time in *A. leiocarpa*) has already been described to have *in silico* replication inhibitory activity against Ebola virus (EBOV) and influenza A (IAV) and B (IBV) viruses.^54,55^ Procyanidin B2 has also been associated with antiviral activity against herpes simplex virus (HSV2) and coxsackie B enterovirus (CVB3).^56^ In addition, procyanidin C (**3**, **5**), first reported in *A. leiocarpa*, has no evidence of antiviral activity, but procyanidin C2 has been described to have anti-inflammatory activity commonly associated with early stages of viral infection.^57^

Epicatechin (**4**), first described in *A. leiocarpa*, has antiviral activity *in vitro* against MAYV and human immunodeficiency virus 1 (HIV-1). In addition, epicatechin appears to be involved in the prevention of influenza A virus (IAV) and Ebola virus (EBOV) infection.^54,58,59^ Homoorientin (**6**), also known as isoorientin, was observed for the first time in this species. This compound has shown antiviral activity *in silico* assays where it interacts with the RBD portion of the spike protein of variants of the SARS-CoV-2 pseudovirus and blocks the binding of SARS-CoV-2 to angiotensin-converting enzyme 2 (ACE2), thereby preventing viral entry into the cell. Other *in vitro* studies show that homoorientin is active against respiratory syncytial virus (RSV).^46,60^

Isovitexin 2-O-arabinoside (**7**) and isovitexin (**8**) are reported for the first time in this species. Isovitexin 2-O-arabinoside has not been reported to have antiviral activity in the literature, but it is related to the compound isovitexin, which showed *in silico* antiviral activity against SARS-CoV-2 by binding to the ACE2 receptor better than existing antiviral drugs, thus preventing the virus from entering the cell. Isovitexin was also active in inhibiting the replication of hepatitis B virus (HBV) DNA.^61,62^ The compound 2-O-rhamnosyl-swertisin (**9**) was observed for the first time in this species. In the scientific literature, this compound is not associated with any activity against viral diseases, but there are *in vivo* studies in animal model that conclude an antinociceptive effect acting in the control of persistent and chronic pain, a persistent symptom in diseases caused by CHIKV.^63^ In addition, 2-O-rhamnosyl-swertisin is related to swertisin, which has *in vitro* and *in vivo* activity against hepatitis B virus (HBV).^64^

(Neo)­astilbin (**10**), astilbin (**11**), and (iso)­astilbin (**12**) are isomers, and the compound astilbin has a variety of biological activities, particularly studies related to the immune system. For example, astilbin has already been shown to inhibit a SARS CoV-2 protease in an antiviral study.^65,66^ Other properties such as antibacterial, antioxidant and hepatoprotective activity have also been described.^65^ Jaceidin (**19**) is mentioned for the first time in *A. leiocarpa*, and the jaceidin triacetate derivative was reported to have inhibitory activity on the SARS CoV-2 protease Mpro.^67^ Chrysosplenetin (**23**) has not yet been reported in this species. This compound has been investigated for the treatment of enterovirus 71 infections, where it was shown to inhibit viral RNA replication.^67,68^ The compound velutin (**24**) is reported for the first time in this species, this compound has been shown to inhibit hepatitis B virus (HBV) *in vitro* in a dose-dependent manner.^69^ Ayanin (**26**), isolated from *A. leiocarpa* by Filho and Gottlieb,^13^ showed positive results against respiratory syncytial virus (RSV), which causes respiratory infections in neonates and young children.^70^

The compound 4′,5,7-trihydroxy-3,6-dimethoxyflavone (**16**) and Eupatilin (**21**) are mentioned for the first time in this species. 4′,5,7-trihydroxy-3,6-dimethoxyflavone (**16**) has activity against *Leishmania amazonensis*.^71^ Eupatilin (**21**) has no reported antiviral activity, but has anti-inflammatory, antioxidant, anticancer, neuroprotective and cardioprotective properties.^72^ Oxyayanin-A (**22**) has already been isolated and reported in *A. leiocarpa*,^13^ and this substance has antitumor activity.^73^ The compounds 5-O-demethylapulein (**14**), apulein (**17**), 5-O-methyloxyayanin-A (**18**), apuleidin (**20**), and apuleirin (**25**) were isolated from *A. leiocarpa* by Filho and Gottlieb,^13^ but at the time of writing no studies are reporting the biological activities of these compounds.

## 5 Conclusions

The present study demonstrated that extracts obtained from *A. leiocarpa* contain a chemically diverse profile, including proanthocyanidins, flavan-3-ols, flavones, flavanones, methoxylated flavonols, and terpenoids, as revealed by LC–MS/MS-based metabolomic analysis. Among the evaluated samples, the ethanolic bark extract (EEBAl) exhibited the most pronounced antiviral activity, particularly against Oropouche virus (OROV), with a favorable selectivity profile. The observed antiviral effects are discussed in light of the relative abundance and chemotaxonomic relevance of annotated metabolites—such as luteolin (**13**), isoluteolin (**15**), 2-O-*p*-coumaroyl alphitolic acid (**27**), and betulinic acid (**28**), which have been previously associated with extract-level antiviral activity in other viral models. However, the antiviral activity observed in this study is attributed to the extract as a whole, and potential contributions of individual compounds should be considered hypothesis-generating rather than causal. In addition, both ethanolic and aqueous extracts derived from bark and leaves showed antiviral effects against ZIKV, MAYV, OROV, and CHIKV, supporting the broad antiviral potential of *A. leiocarpa*. Overall, these findings highlight *A. leiocarpa*, particularly the ethanolic bark extract, as a promising source of antiviral agents. Future studies will focus on bioassay-guided fractionation and mechanistic investigations to identify active constituents and elucidate their modes of antiviral action.

## Supplementary Material



## Notes

Detailed metabolite characterization, including fragments (MS/MS) explanation and mechanisms (PDF)
