# Supplementary material for: Ethnopharmacology- and Chemotaxonomy-Guided Evaluation of Apuleia leiocarpa Bark and Leaf Extracts against Oropouche, Mayaro, Chikungunya, and Zika Viruses
Source: ACS Omega. 2026 Mar 12;11(11):17472–83. doi: 10.1021/acsomega.5c10741 (PMC13019222; doi:10.1021/acsomega.5c10741)
Supplement: Supplementary file 1 [file ao5c10741_si_002.pdf]

# Ethnopharmacology- and Chemotaxonomy-Guided Evaluation of *Apuleia leiocarpa* Bark and Leaf Extracts Against Oropouche, Mayaro, Chikungunya, and Zika Viruses

Maria Rosilda Valente de Sarges[1,2], Maria Clara Machado Oliveira[5], Mariana Pereira de Carvalho[5], Marília Bueno da S. Menegatto[4], Ariane Coelho Ferraz[4], Sônia das Graças Santa Rosa Pamplona[1,3], Wandson Braamcamp Pinheiro[1,3], Cintia Lopes B. Magalhães[4,6], Milton Nascimento da Silva[1,3], Paulo Wender P. Gomes[1,3], Consuelo Yumiko Yoshioka e Silva[1,2,*]

[1]Laboratory of Liquid Chromatography, Federal University of Pará, Belém 66075-110, Brazil;
[2]Institute of Health Sciences, Pharmaceutical Sciences Post-Graduation Programme, Federal University of Pará, Belém 66075-110, Brazil;
[3]Institute of Exact and Natural Sciences, Federal University of Pará, Belém 66075-110, Brazil;
[4]Biology Sciences Post-Graduation Program, Federal University of Ouro Preto, Ouro Preto, Minas Gerais, Brazil
[5]School of Pharmacy, Federal University of Ouro Preto, Ouro Preto, Minas Gerais Brazil
[6]Biotechnology Post-Graduation Program, Federal University of Ouro Preto, Ouro Preto, Minas Gerais, Brazil

**Correspondence**

*Corresponding author: Consuelo Y. Y. e Silva, , Institute of Health Sciences, Federal University of Pará, Augusto Corrêa, 01, Guamá, Belém 66075-110, Pará, Brazil.
*E-mail addresses:* cyoshioka@ufpa.br

## SUPPORTING INFORMATION

Compounds **1** (*m/z* 577.1353) and **2** (*m/z* 577.1349) were characterised as procyanidin B isomers. Both compounds showed the presence of fragments at *m/z* 125, 161, 205, 245, 289, 339, 407 and 425. In accordance with the proposed fragmentation for both compounds, the *m/z* 425 fragment is attributed to a retro-Diels-Alder (RDA) cleavage in the flavan-3-ol C-ring (-152 Da), followed by a loss of water (18 Da). This is a common occurrence in this flavonoid subclass and results in the formation of the *m/z* 407 fragment. Conversely, the fragment at *m/z* 289 is indicative of a terminal (epi)catechin moiety generated by the QMupper reaction, which allows interflavan-3-ols to be cleaved. Finally, the fragment at *m/z* 125, designated trihydroxybenzoic, is a relatively

common occurrence and is typically produced by the loss of the B-ring and part of the C-ring in flavan-3-ols.[27,28] Therefore, these data allow us to rationalise that compounds **1** and **2** are isomers of procyanidins B with stereochemical differences and most likely epimers.

Compounds **3** and **5** (*m/z* 865.1993 and *m/z* 865.1995) showed the presence of fragments with *m/z* values of 125, 287, 289, 407, 425, 575 and 695. The proposed fragment *m/z* 695 can be attributed to an RDA cleavage in the C ring of the first trimer extension, followed by the loss of $H_2O$. The *m/z* 577 fragment can be attributed to the cleavage of the interflavan-3-ol bond. Subsequently, the *m/z* 425 fragment is derived from an RDA cleavage in the C ring, which is also capable of losing $H_2O$, resulting in the formation of the *m/z* 407 fragment. The *m/z* 289 fragment is indicative of the (epi)catechin terminus. Finally, the *m/z* 287 fragment can be generated from the *m/z* 577 ion by a QMupper reaction described above. The fragment at *m/z* 125 is identical to that already discussed for compounds **1** and **2**. These fragmentation proposals suggest that compounds **3** and **5** are isomers of procyanidin C.[29]

Compound **4** (*m/z* 579.1494) was identified as a dimerised form of (epi)catechin $(2M-H)^-$. The resulting fragments were *m/z* 109, 125, 151, 179, 203, 205, 221 and 245. The proposed fragmentation provides a rationale for the hypothesis that the *m/z* 245 fragment corresponds to the loss of $C_2H_4O$ in the C ring. The *m/z* 205 fragment is indicative of a cleavage event in the A ring, accompanied by the loss of $C_4H_6O_2$. The *m/z* 179 fragment results from the loss of $C_6H_6O_2$. The *m/z* 203 fragment corresponds to the loss of $C_2H_2O$ by the *m/z* 245 fragment. Based on these observations, it can be concluded that the compound in question is most likely (epi)catechin.[30,31] Fragmentation of compound **6** (*m/z* 447.0930) was observed at *m/z* 133, 163, 175, 285, 297, 311, 327, 339, 357 and 429. The *m/z* 429 fragment indicates the loss of a water molecule resulting in a mass shift of 18 Da. The *m/z* 357 and 327 fragments, which are both -90 Da and -120 Da respectively, are thought to indicate the cleavage of a hexose. The *m/z* 285 fragment may be due to the total loss of a hexose (162 Da). Consequently, this compound has been named homoorientin.[32,33]

The fragmentation pattern of compound **7** (*m/z* 563.1402) gave the following fragments: *m/z* 293, 311, 323, 413 and 431. The *m/z* 431 fragment can be attributed to the loss of a rhamnose deoxy sugar (146 Da). The *m/z* 413 fragment theoretically corresponds to the loss of a water molecule (18 Da) from the *m/z* 431 fragment. Consequently, based on these fragmentation

characteristics, compound **7** was identified as a trihydroxyflavone known as isovitexin 2-O-arabinoside.[34,35] The fragmentation pattern of compound **8** (*m/z* 431.0978) gave fragments *m/z* 269, 283, 311, 323 and 341. The *m/z* 311 fragment may be the result of the cleavage of part of a glycone (120 Da), with the subsequent fractions involved in the loss of the aglycone fraction. A review of the literature showed that *m/z* 431.0978 is consistent with the fragmentation pattern of isovitexin.[36,37]

Compound **9** (*m/z* 591.1711) showed fragmentation at *m/z* 283, 295, 324, 325, 427, 445 and 471. The *m/z* 471 fragment is probably derived from the cleavage of part of a hexose (120 Da), while the *m/z* 445 fragment is the result of cleavage at another hexose position (146 Da). This fragment theoretically corresponds to the compound swertisin (456 Da). The *m/z* 427 fragment indicates the complete loss of a hexose (164 Da). The *m/z* 325 fragment is thought to originate from the degradation of another sugar (120 Da). Consequently, compound **9** was annotated as 2-O-rhamnosyl-swertisin.[38]

Ions **10** (*m/z* 449.1098), **11** (*m/z* 899.2274) and **12** (*m/z* 449.1090) showed the presence of fragments with *m/z* values of 107, 125, 151, 178, 285 and 303 respectively. The fragment at *m/z* 303 indicates the loss of part of the hexose ($C_6H_{10}O_4$) (146 Da). The fragment at *m/z* 285 results from the total loss of the hexose (164 Da), indicating the presence of the aglycone, which in this case is defined as a flavanone due to the presence of a carbonyl group at C-4. Fragment *m/z* 151 indicates the loss of $C_{14}H_6O_7$ (298 Da), confirming the presence of the A and C rings of the flavanone. In addition, the smaller fragments, including *m/z* 178, 125 and 107, are consistent with the findings presented in the peer-reviewed literature.[39] These fragmentation pathways are typical for this flavonoid subclass. In light of the above evidence, it is reasonable to propose that compounds **10**, **11**, and **12** correspond to (neo)astilbin, astilbin, and (iso)astilbin,[40] although this conclusion is not without reservations. It is clear that there are potential stereoisomers, such as neoisoastilbin, which could influence the final characterisation of these compounds. Therefore, the most accurate definition would require the use of standards for these four compounds or their isolation and comprehensive elucidation by nuclear magnetic resonance experiments using both $^{1}H$ and $^{13}C$.

Fragmentation of compound **13** (*m/z* 285.0393) gave the following fragments: *m/z* 107, 133, 151, 175, 199 and 217. Compound **15** (*m/z* 285.0400) also showed fragmentation of 151, 199,

217, 241 and 257. The possibilities for fragmentation of these *m/z* values include breaks attributed to a C ring. For example, the *m/z* 267 fragment could be formed by the loss of a water molecule (18 Da), the *m/z* 257 fragment by the loss of CO (28 Da) and the *m/z* 241 fragment by the loss of $CO_2$ (44 Da). In another part of the molecule, in ring A, there may be scissions giving rise to *m/z* 217 due to the loss of $C_3O_2$ (68 Da). This then leads to the formation of the *m/z* 175 fragment, which is formed by the loss of $C_2H_2O$ (110 Da) from the above fragment. It is reasonable to assume that the *m/z* 151 and *m/z* 133 fragments correspond to the cleavage of the molecule by retro-Diels-Alder (RDA) cleavage in the C ring. On the basis of these fragmentation characteristics, it can be postulated that compounds **13** and **15** correspond to luteolin and (iso)luteolin isomers, respectively.[41,42]

Compound **16** (*m/z* 329.0659) had the fragments *m/z* 199, 243, 271, 299, 314. This compound was previously annotated as 4',5,7-trihydroxy-3,6-dimethoxyflavone. Fragment *m/z* 314 may be formed by loss of $CH_3$ (15 Da). Fragment *m/z* 299 may be related to the loss of $2CH_3$ (30 Da). Fragment *m/z* 271 may result from the loss of $C_3H_6O$ (58 Da). A possible explanation for the *m/z* 243 fragment is the loss of $C_4H_6O_2$ (86 Da), then the loss of $CO_2$ (44 Da) from the *m/z* 243 fragment produces the *m/z* 199 fragment. Compound **18** (*m/z* 373.0926) had fragments at *m/z* 165, 207, 287, 297, 315, 326, 343, 358 and compound **23** (*m/z* 373.0917) fragmented at *m/z* 257, 285, 300, 315, 328, 343, 358. The *m/z* 358 fragment may have originated from $CH_3$ loss (15 Da). The *m/z* 343 fragment is probably due to the loss of $2CH_3$ (30 Da). The *m/z* 315 fragment may be due to the loss of $C_3H_6O$ (58 Da). Compounds **18** and **23** were thus annotated as 5-O-methyloxyayanin-A and chrysosplenetin, respectively.[43] Compound **14** (*m/z* 389.0868) was annotated as 5-O-demethylapulein. Compound **17** (*m/z* 403.1024) was annotated as apulein. Compound **25** (*m/z* 403.1035) was annotated as apuleirin. These compounds were annotated based on the study by Braz Filho and Gottlieb (1971) [44] who isolated and reported the presence of 5-O-demethylapulein, apulein, apuleirin in *A. leiocarpa*.

The mass spectrum of substance **19** (*m/z* 359.0763) exhibited fragment ions *m/z* 258, 286, 301, 312, 314, 329 and 344, which are consistent with the data presented in the scientific literature for the annotation of the compound jaceidin.[45] Compound **20** (*m/z* 359.0756) showed the following fragment ions: *m/z* 299, 312, 314, 327, 344. Fragmentation of compound **22** (*m/z* 359.0760) occurred at *m/z* 221, 243, 267, 289, 299, 311, 327 and 344. It is hypothesised that the fragment at

*m/z* 344 is the result of the loss of a $CH_3$ group (15 Da). Compounds **20** and **22** have been named apuleidin and oxyayanin-A respectively. These isomers were previously isolated from the species *A. leiocarpa*.[44]

Compound **21** (*m/z* 343.0815) gave the following fragments: *m/z* 226, 242, 270, 285, 298, 313, 328 and the compound itself. The **26** (*m/z* 343.0813) gave the following fragment ions: *m/z* 186, 198, 214, 226, 242, 254, 270 and 298. It is reasonable to conclude that the *m/z* 328 fragment indicates the loss of a methyl group ($CH_3$ - 15 Da). The *m/z* 313 fragment can be attributed to the loss of two methyl groups (30 Da). The possible loss of three methyl groups (45 Da) gives rise to the *m/z* 298 fragment in the compounds. Compound **21** was tentatively identified as eupatilin, while compound **26** was designated as ayanin. The latter was previously isolated from the species in a separate study.[44,46] Compound **24** (*m/z* 313.0710) showed the following fragment ions: *m/z* 255, 270, 283 and 298. The loss of one methyl group ($CH_3$ - 15 Da) results in the generation of the *m/z* 298 fragment. Subsequently, the loss of two methyl groups ($2CH_3$ - 30 Da) results in the formation of the *m/z* 283 fragment. The *m/z* 255 fragment can be attributed to the loss of $C_3H_6O$ (58 Da). Based on these observations, compound **24** was named velutin.[47]

Mass spectrometric analysis of compound **27** (*m/z* 617.3846) revealed the presence of the following fragments: *m/z* 117, 133, 145, 161, 423, 439, 455 and 573. Fragment *m/z* 573 can be attributed to the loss of $CO_2$ (44 Da). Fragment ion *m/z* 145 is associated with the coumaroyl fraction resulting from the cleavage of the molecule (loss of 472 Da). In accordance with the data presented in the literature spectra, this compound (**27**) has been designated as 2-O-p-coumaroyl alphitolic acid.[48] Compound **28** (*m/z* 455.3529) showed fragment ions *m/z* 207, 391, 407, 437. The *m/z* 437 fragment is associated with the loss of $H_2O$ (18 Da), followed by the loss of $CH_2O_2$ (46 Da) to form the *m/z* 391 fragment. The *m/z* 407 ion is reported to be due to the loss of $CH_2O$ and $H_2O$ (48 Da), while the *m/z* 207 fragment ($C_{14}H_{23}O$) corresponds to cleavage by the retro-Diels-Alder (RDA) reaction in the C ring of a triterpene molecule. Based on these features and the report of a previous study on *A. leiocarpa*, compound **28** was annotated as betulinic acid.[49-51] Compounds **29** (*m/z* 603.4048) and **30** (*m/z* 603.4057) with different retention times showed fragments of *m/z* 133, 135, 161, 179. These fragments are consistent with the analysis of existing studies in literature and show the characteristics of triterpene compounds, so it is inferred that these *m/z* can be referred to as 3-O-caffeoyl-betulin and 3-O-caffeoyl-betulin isomers, respectively.[52,53]

**SEÇÃO 2.2 – LC–MS/MS analysis**

The scan time was 0.1 s, and the charge states were +2, with a tolerance window of ± 0.2 Da and a peak extraction tolerance of 2 Da. The deisotope tolerance was ± 3 Da and the deisotope extraction tolerance was 6 Da.

The source and desolvation temperatures were set at 150 °C and 300 °C, respectively. The cone and desolvation gas flow rates were 50 L/h and 800 L/h, respectively. The capillary voltage was 3.0 kV and the cone voltage was 40 V.
